# Abortive Herpes Zoster Ophthalmicus in a High-Risk Pregnancy Following Ultra-Early Valacyclovir Therapy: A Case Report

**DOI:** 10.7759/cureus.107145

**Published:** 2026-04-16

**Authors:** Donna May A Sarenas

**Affiliations:** 1 Dermatology, Southern Philippines Medical Center, Davao City, PHL

**Keywords:** abortive herpes zoster, anti-phospholipid antibody syndrome (aps), herpes zoster ophthalmicus, high-risk pregnancy, valacyclovir

## Abstract

Herpes zoster ophthalmicus (HZO) is a manifestation of varicella-zoster virus reactivation involving the ophthalmic division of the trigeminal nerve and carries a risk of significant ocular complications. Early antiviral therapy may alter the clinical course of infection and occasionally produce atypical or abortive presentations. This report describes the case of a 34-year-old primigravid woman at 33 weeks of gestation with primary antiphospholipid antibody syndrome on anticoagulation therapy who presented with acute severe burning pain over the right forehead and scalp followed by grouped erythematous papules along the ophthalmic dermatome. Oral valacyclovir was initiated within eight hours of rash onset. The lesions evolved into erythematous, edematous plaques without vesiculation, pustulation, or crusting and resolved completely without ocular involvement or postherpetic neuralgia. The patient subsequently delivered a healthy neonate at term without complications attributable to maternal infection or antiviral therapy. This case demonstrates that ultra-early antiviral therapy may attenuate viral replication and modify lesion morphology and is associated with an abortive non-vesicular presentation. Recognition of this variant is important to ensure timely diagnosis and management, particularly in a high-risk pregnancy.

## Introduction

Herpes zoster arises from reactivation of latent varicella-zoster virus (VZV) within sensory ganglia and typically manifests as a painful unilateral vesicular eruption distributed along a dermatome [1,2]. In contrast to primary varicella infection, which presents as a generalized vesicular eruption, herpes zoster reflects reactivation of latent virus following prior infection [1,2]. When reactivation involves the ophthalmic division of the trigeminal nerve, the condition is referred to as herpes zoster ophthalmicus (HZO), which represents approximately 10-20% of all herpes zoster cases [3,4]. HZO is clinically significant because ocular involvement develops in up to 50% of cases and may result in keratitis, uveitis, retinitis, and optic neuropathy, potentially leading to permanent visual impairment if not promptly recognized and treated [3,5].

Herpes zoster during pregnancy is relatively uncommon and is generally associated with favorable maternal and fetal outcomes with minimal risk of congenital infection compared with primary varicella infection [6]. Nevertheless, timely recognition and management remain essential, particularly in high-risk pregnancies or when the ophthalmic dermatome is involved. Antiviral therapy initiated within 72 hours of rash onset has been shown to reduce viral replication, shorten disease duration, and decrease the likelihood of complications such as postherpetic neuralgia [2,7].

The timing of antiviral therapy may also influence the morphologic evolution of cutaneous lesions. Early administration has been associated with a reduction in new vesicle formation and more rapid lesion resolution, suggesting that suppression of viral replication at an early stage may alter the typical disease course [2,8-10]. In some cases, this may result in attenuated or atypical presentations that do not progress to the classic vesicular phase. 

Abortive herpes zoster refers to a modified clinical presentation in which typical vesicle formation is absent or minimal, often manifesting as erythematous papules or plaques without progression to vesiculation [2,9,10]. This phenomenon is thought to result from early suppression of viral replication, most commonly associated with prompt antiviral therapy.

This report describes a case of HZO in a high-risk pregnancy complicated by antiphospholipid antibody syndrome (APS), in which valacyclovir therapy was initiated within eight hours of rash onset, representing an ultra-early antiviral intervention, well before the conventional 72-hour treatment window recommended for herpes zoster management [2,7]. This was associated with an abortive, non-vesicular presentation and the absence of complications.

## Case presentation

A 34-year-old primigravid woman at 33 weeks of gestation with primary APS, maintained on prophylactic tinzaparin, presented with an acute onset of severe burning pain localized to the right forehead and scalp. The pain was neuropathic in quality and rated at 8/10 on the Numeric Rating Scale (NRS), consistent with typical acute herpes zoster-associated pain and raising early clinical suspicion for herpes zoster. Mild ocular discomfort was also reported.

Within 24 hours of symptom onset, a few grouped erythematous papules developed over the right forehead in the ophthalmic (V1) dermatomal distribution of the trigeminal nerve. The pain increased in severity and radiated to the right scalp, cheek, ear, and ipsilateral maxillary teeth region.

**Figure 1:**
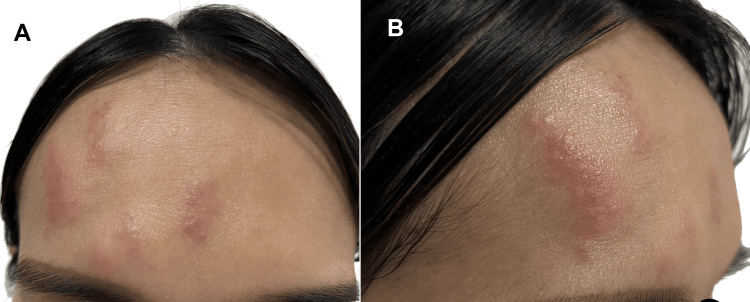
(A) Frontal view showing erythematous, edematous papules and plaques localized to the right forehead in the ophthalmic (V1) dermatomal distribution. (B) Close-up view demonstrating papules and plaques without vesiculation, pustulation, or crusting. The absence of vesicle formation is clinically significant and supports an abortive, non-vesicular presentation of herpes zoster.

Oral valacyclovir (1 g three times daily) was initiated approximately eight hours after rash onset. Over the subsequent 48 hours, additional lesions appeared along the same dermatome. However, instead of progressing to vesicles, the lesions evolved into erythematous, edematous papules and plaques (Figure 1) without vesiculation, pustulation, or crusting. A limited number of lesions subsequently appeared on the upper eyelid and nasal bridge, consistent with involvement of the nasociliary branch of the ophthalmic nerve.

Ophthalmologic evaluation demonstrated mild periocular tenderness without evidence of keratitis, conjunctivitis, uveitis, or visual impairment. Neuralgic symptoms fluctuated between days 5 and 7, characterized by intermittent shooting pain involving the scalp and jaw. As the pain subsided, pruritus became more prominent. By day 9, the pain had largely resolved, and the cutaneous lesions gradually regressed without scarring. Residual erythema resolved completely over the following two weeks. No disseminated lesions were observed.

The diagnosis was made clinically based on the characteristic dermatomal neuropathic pain followed by a unilateral eruption in the ophthalmic distribution, a well-recognized diagnostic pattern in herpes zoster. Confirmatory testing such as PCR was not performed, as it would not have altered immediate management, and early antiviral therapy was prioritized given the risk of ocular involvement. 

The pregnancy progressed to 37 weeks of gestation, at which point a cesarean delivery was performed for arrest of dilation. The neonate required brief phototherapy for hyperbilirubinemia but had no complications attributable to maternal herpes zoster or antiviral therapy. At eight-month follow-up, both mother and infant remained well without sequelae (Table 1).

**Table 1:** Timeline of clinical course and management. NRS: Numeric Rating Scale; TID: three times daily.

Time point	Clinical events
Day 0	Onset of severe neuropathic pain over right forehead and scalp (8/10 NRS)
Day 1 (within 24 hours)	Development of grouped erythematous papules in V1 distribution
~8 hours after rash onset	Initiation of oral valacyclovir (1 g TID)
Days 2–3	Lesions increased but evolved into erythematous papules and plaques without vesiculation
Days 5–7	Fluctuating neuralgic pain; onset of pruritus
Day 9	Resolution of pain; regression of lesions
2 Weeks	Complete resolution of erythema
37 Weeks of gestation	Cesarean delivery; healthy neonate
8-Month follow-up	No sequelae in mother or infant

## Discussion

Herpes zoster represents reactivation of latent VZV within sensory ganglia and most frequently involves thoracic dermatomes [1,2]. Involvement of the ophthalmic branch of the trigeminal nerve results in HZO, which accounts for approximately 10-20% of cases and carries a risk of ocular complications [3,4]. Population-based studies have demonstrated an increasing incidence of HZO in recent decades [5].

While herpes zoster classically presents as a vesicular dermatomal eruption, atypical manifestations are increasingly recognized. These include non-vesicular presentations as well as zoster sine herpete, in which patients develop characteristic dermatomal neuropathic pain without visible cutaneous lesions [9,10]. This entity, first described by Lewis in 1958 [11], has since been supported by modern diagnostic techniques, including polymerase chain reaction and serologic testing, confirming that VZV reactivation may occur in the absence of vesicular eruptions [2,9,10]. Awareness of these variants is important, as delayed recognition may result in missed opportunities for early antiviral therapy.

The differential diagnosis of non-vesicular dermatomal eruptions includes herpes simplex infection, contact dermatitis, impetigo, insect bite reactions, and early cellulitis. However, the presence of prodromal neuropathic pain, strict unilateral dermatomal distribution, and subsequent evolution of lesions strongly supported herpes zoster in this case [1,2].

Acute herpes zoster is frequently preceded by moderate to severe neuropathic pain, often rated between 6 and 8 on the NRS, and may reach higher intensities in cases involving the ophthalmic dermatome [2,7]. In this patient, the presence of severe burning pain rated at 8/10, with a dermatomal distribution, heightened early clinical suspicion for herpes zoster despite the absence of vesicles. Recognition of this characteristic pain pattern is particularly important in atypical or abortive presentations, as it may facilitate early diagnosis and prompt initiation of antiviral therapy prior to full cutaneous expression.

Although polymerase chain reaction testing can provide definitive confirmation of VZV infection, diagnosis is frequently made on clinical grounds, particularly when dermatomal neuropathic pain precedes a unilateral eruption [1,2]. In this case, early antiviral therapy was prioritized to reduce the risk of ocular complications, given the involvement of the ophthalmic dermatome, consistent with current guideline recommendations emphasizing prompt treatment in high-risk cases [3,7].

Systemic antiviral therapy forms the mainstay of treatment for herpes zoster. Current guidelines recommend initiation within 72 hours of rash onset to reduce viral replication, shorten disease duration, and lower the risk of complications, including postherpetic neuralgia and ocular involvement [2,3,7]. Early treatment is particularly important in HZO, as prompt antiviral therapy can limit lesion progression and reduce vision-threatening sequelae [3]. Randomized trials have demonstrated that antiviral therapy reduces the severity and duration of acute symptoms [8,12].

In this case, valacyclovir was initiated approximately eight hours after rash onset, considerably earlier than the treatment windows evaluated in most clinical trials. Ultra-early antiviral therapy may have contributed to suppression of viral replication before vesicle formation, potentially resulting in attenuated lesion morphology and preventing progression to the classic vesicular stage [2,8,12]. Such early intervention is consistent with observations that prompt antiviral initiation reduces both the severity and duration of lesions and may account for atypical or abortive presentations [10]. In this patient, the eruption consisted of erythematous papules and plaques without progression to vesiculation or crusting, consistent with an abortive clinical course.

Herpes zoster during pregnancy is relatively uncommon, with an estimated incidence of 0.1-0.7 per 1,000 pregnancies [6,13]. Compared with primary varicella infection, reactivation of VZV in pregnancy is generally associated with favorable maternal and fetal outcomes, as transplacental transmission is rare and congenital varicella syndrome is typically not a concern [6,13]. Rather than representing a state of generalized immunosuppression, pregnancy involves complex immunologic adaptations that allow fetal tolerance while preserving key components of cell-mediated immunity necessary for control of latent viral infections such as VZV [14,15]. This preservation likely contributes to the low incidence of herpes zoster in pregnant women and the generally favorable maternal and fetal outcomes observed [6]. In this patient, despite the presence of pregnancy and autoimmune comorbidity, the clinical course remained localized and uncomplicated.

Another important consideration in this case is APS, an autoimmune thrombophilic disorder associated with recurrent thrombosis and adverse pregnancy outcomes [16]. Management typically includes anticoagulation therapy during pregnancy to reduce maternal and placental thrombotic risk [17]. Although APS does not directly predispose to VZV reactivation, the presence of multiple comorbidities underscores the importance of early recognition and prompt antiviral therapy in high-risk patients, in whom complications may carry greater clinical significance.

To the author’s knowledge, this case suggests that initiation of antiviral therapy earlier than the traditionally recommended 72-hour window may influence the clinical expression of herpes zoster. The absence of vesiculation and rapid resolution without complications suggests that viral replication may have been suppressed before full cutaneous expression. Recognition of such abortive presentations is clinically important, as the lack of classic vesicles may delay diagnosis if herpes zoster is not considered in patients presenting with dermatomal pain and non-vesicular cutaneous findings. 

This report has several limitations. The diagnosis was made clinically without virologic confirmation, and as a single case report, the findings are hypothesis-generating and do not establish causation. Further studies are needed to clarify the potential impact of ultra-early antiviral therapy on disease morphology.

## Conclusions

This case describes an atypical, abortive presentation of HZO in a high-risk pregnancy complicated by APS. Initiation of valacyclovir within hours of rash onset was associated with a non-vesicular clinical course and the absence of ocular complications or postherpetic neuralgia. This report suggests that ultra-early antiviral therapy may be associated with attenuation of viral replication and modification of lesion morphology, although this observation is based on a single case and requires further study. Recognition of such abortive presentations is important, as the absence of classic vesicles may delay diagnosis and treatment if herpes zoster is not considered in patients presenting with dermatomal neuropathic pain and atypical cutaneous findings. Early clinical suspicion and prompt treatment remain critical, particularly in cases involving the ophthalmic dermatome.
